# Two new species of spotted *Hypancistrus* from the Rio Negro drainage (Loricariidae, Hypostominae)

**DOI:** 10.3897/zookeys.552.5956

**Published:** 2016-01-13

**Authors:** Milton Tan, Jonathan W. Armbruster

**Affiliations:** 1 Department of Biological Sciences, 101 Life Sciences Building, Auburn University, AL 36849, USA.

**Keywords:** Siluriformes, Guyana, Brazil, taxonomy, suckermouth armored catfish, Neotropics

## Abstract

Two new species, *Hypancistrus phantasma* and *Hypancistrus margaritatus*, are described based on material from the Rio Negro drainage. Both species are distinguished from congeners by unique color patterns. *Hypancistrus phantasma* is described from the Rio Uaupes and differs from congeners by having a tan body with small dark spots (vs. dark with light spots or with saddles or stripes). *Hypancistrus margaritatus* is described from the Takutu River and differs from congeners by having densely-packed light spots on a dark brown background, with spots about the size of the nasal aperture (vs. sparse light spots either smaller or larger than the nasal aperture, or brown to black spots, saddles, or stripes).

## Introduction

*Hypancistrus* is a small genus comprising six species of loricariid catfishes in the tribe Ancistrini of the subfamily Hypostominae: *Hypancistrus zebra* Isbrücker and Nijssen (1991), the type species from the Rio Xingu; *Hypancistrus inspector* Armbruster (2002) from the Rio Negro; and *Hypancistrus contradens* Armbruster et al. (2007), *Hypancistrus debilittera* Armbruster et al. (2007), *Hypancistrus furunculus* Armbruster et al. (2007), and *Hypancistrus lunaorum* Armbruster et al. (2007), all from the Rio Orinoco.

*Hypancistrus* can be distinguished externally from most other hypostomines by having larger and fewer teeth on the dentary relative to the teeth on the premaxilla. Different numbers of teeth on the dentary and premaxilla are found in many loricariids, so this character is not always diagnostic (Armbruster 2002). The genus is diagnosed by the unique presence of a wide anterior separation between the metapterygoid and the lateral ethmoid, and a sharply angled adductor palatini crest. It also lacks the lateral wall of the metapterygoid channel (Armbruster 2002).

Species of *Hypancistrus* are best distinguished from each other by their color pattern and cannot be separated by traditional morphometrics used in loricariids (Armbruster et al. 2007). Numerous undescribed forms remain, and there is considerable variation in color patterns (Armbruster et al. 2007). Undescribed forms of *Hypancistrus* (and other loricariids) are assigned L-numbers by the aquarium fish magazine DATZ that are widely used by aquarists, and some of these forms are discussed. Here we describe two new species of *Hypancistrus* with spotted color patterns.

## Methods

Measurements and counts follow Armbruster (2003) with additional counts for lateral plate series from Armbruster et al. (2007). Anatomical nomenclature follows Schaefer (1987) and Geerinckx et al. (2007) for skeletal characteristics, Schaefer (1997) for names of plate rows, Douglas et al. (2002) for the term ‘iris operculum,’ and Armbruster et al. (2007) for the term ‘nasal aperture.’ We define minimum interspot distance as the minimum distance between spots measured. Midlateral spots were selected from behind the head to the base of the caudal peduncle for measurement, and minimum interspot distance is given as a ratio over spot diameter. Institutional abbreviations are as listed in Sabaj Pérez (2014).

## Systematics

### Hypancistrus phantasma sp. n.

Taxon classificationAnimaliaSiluriformesLoricariidae

http://zoobank.org/D20CFAAD-CE01-4F2F-99D9-7E36B1D6C0F3

Figure 1

#### Holotype.

MZUSP 116531, 123.3 mm SL, Amazon Basin, Rio Negro drainage: Rio Uaupes, Taracua, 0.1°N, 68.46667°W, 14 Feb 1924, D. Melin and A. Vilars.

#### Paratypes.

NRM 16880, 3, 92.5–105.46 mm SL, same data as holotype; NRM 39344, 1, 101.3 mm SL, same locality as holotype, 19 Feb 1924, same collectors as holotype.

#### Diagnosis.

*Hypancistrus phantasma* can be distinguished from congeners by a color pattern consisting of a tan base coloration with black spots vs. a color pattern consisting of a dark base coloration and light spots (as in *Hypancistrus contradens*, *Hypancistrus inspector*, *Hypancistrus lunaorum* and *Hypancistrus margaritatus*) or a color pattern consisting of saddles, vermiculations, bands, or stripes (as in *Hypancistrus furunculus*, *Hypancistrus debilittera*, and *Hypancistrus zebra*).

#### Description.

Morphometric data given in Table 1. Counts are variation observed in paratypes. Largest specimen examined 123.3 mm SL. Ventral surface from anus to head largely lacking plates in adults. Small plates present in skin ventral to pectoral girdle, anteromesially to gill opening, and on ventrolateral margins of thorax extending posteriorly from insertion of pectoral-fin spine towards insertion of pelvic-fin spine. Extent of small ventral plates correlated with body size, with larger specimens having larger ventral plated areas with plates ventral to pectoral girdle joining medially, and ventrolateral abdominal plates extending further posteriorly. Lateral plates unkeeled except ventral plate row with moderately elongate odontodes forming one or two keel-like rows on caudal peduncle, and mid-ventral plate row bent above pectoral girdle forming ridge continuous with cleithrum. Cheek plates with 23–49 hypertrophied odontodes. Frontal, infraorbitals, nasal, preopercle, compound pterotic, and suprapreopercle supporting odontodes. Small ridge of opercle covered in smallest paratype (92.5 mm SL), exposed in remaining specimens; if exposed, zero to six odontodes present.

**Table 1.** Morphometric data of new *Hypancistrus* species. Morphometric data of *Hypancistrus phantasma* (n = 5) and *Hypancistrus margaritatus* (n = 1). Morphometric data except standard length are given as percentages of standard length.

	*Hypancistrus phantasma*					*Hypancistrus margaritatus*
	Holotype	Avg	SD	Min	Max	Holotype
SL	123.3	103.3	12.4	92.5	123.3	45.6
Predorsal Length	42.3	42.8	0.4	42.3	43.4	45.5
Head Length	35.0	35.8	0.5	35.0	36.5	38.4
Head-dorsal Length	7.3	7.1	0.4	6.6	7.6	7.5
Cleithral Width	30.6	31.4	0.8	30.6	32.7	31.2
Head-pectoral Length	26.7	25.8	0.8	24.8	26.7	26.6
Thorax Length	23.4	23.0	0.5	22.4	23.6	25.6
Pectoral-spine Length	32.8	34.3	1.6	32.8	36.8	35.7
Abdominal Length	24.6	25.0	0.2	24.6	25.2	24.2
Pelvic-spine Length	26.5	27.3	0.9	26.3	28.5	33.3
Postanal Length	32.4	32.6	0.7	32.1	33.8	34.1
Anal-fin spine Length	10.3	11.7	0.9	10.3	12.6	14.6
Dorsal-pectoral Distance	28.3	28.9	0.5	28.3	29.6	29.5
Dorsal spine Length	32.3	33.8	2.1	32.1	36.7	36.6
Dorsal-pelvic Distance	26.1	25.2	0.8	24.3	26.1	24.1
Dorsal-fin base Length	28.3	27.7	0.6	26.7	28.3	29.0
Dorsal-adipose Distance	16.9	16.5	0.7	15.2	17.1	16.1
Adipose-spine Length	7.1	8.5	0.9	7.1	9.0	8.5
Adipose-upper caudal Distance	13.4	14.3	1.1	13.4	15.8	11.9
Caudal peduncle Depth	11.2	11.0	0.8	9.6	11.7	13.0
Adipose-lower caudal Distance	21.2	22.4	0.8	21.2	23.1	21.0
Adipose-anal Distance	19.4	19.2	0.5	18.3	19.6	19.5
Dorsal-anal Distance	17.4	17.3	0.7	16.5	18.2	15.9
Pelvic-dorsal Distance	28.2	28.1	0.7	27.0	28.9	26.0
Head-eye Length	11.7	12.0	0.3	11.7	12.4	13.8
Orbit Diameter	8.3	8.9	0.5	8.3	9.4	10.0
Snout Length	19.7	19.7	0.3	19.3	20.0	21.0
Internares Width	4.9	5.1	0.5	4.7	5.9	5.5
Interorbital Width	15.1	16.1	0.7	15.1	16.8	17.9
Head Depth	24.8	25.3	0.4	24.8	25.8	26.3
Mouth Length	14.6	15.2	0.4	14.6	15.5	17.4
Mouth Width	12.2	13.3	1.8	11.2	16.1	17.7
Barbel Length	4.0	4.2	0.6	3.7	5.2	3.2
Dentary tooth cup Length	4.0	3.9	0.4	3.4	4.5	2.3
Premaxillary tooth cup Length	2.0	1.8	0.2	1.4	2.0	2.8

Caudal fin emarginate, lower lobe longer than upper. Ventral surface flat. Head tall. Snout short. Vertical through anterior margin of orbit about half-way between vertical through anterior margin of snout and vertical through posteromedial tip of supraoccipital. Head with steep angle in profile, roughly 45° from tip of snout to anterior margin of eye. Body depth increases gradually from anterior margin of eye to dorsal-fin origin, then decreases gradually from dorsal-fin origin to posterior insertion of adipose fin; caudal-peduncle depth increases slightly from insertion of adipose fin to origin of caudal fin.

Supraorbital crest low. Orbital opening oriented at less than 45°from sagittal plane. Interorbital isthmus between supraorbital crests flat. Supraoccipital crest low. Eye large with iris operculum. Gill opening restricted in *L*-conformation, with half of opening vertical and opening laterally and half of opening horizontal and opening ventrally.

Dorsal-fin spine short; depressed dorsal fin reaching anterior edge of or slightly beyond preadipose plate. Depressed pectoral-fin spine reaching beyond base of pelvic-fin rays; depressed pelvic-fin spine reaching beyond posterior insertion of anal fin. Dorsal fin II,7; caudal fin I,14,I; dorsal procurrent caudal-fin spines four to five, four in holotype; ventral procurrent caudal-fin spines four to five, four in holotype; anal fin i3–4 (one paratype examined with 3 anal-fin rays); pectoral fin I,6; pelvic fin i,5. Fin spines and rays supporting odontodes. Odontodes more elongate distally on pectoral-fin spine and ventrally on pelvic-fin spine than at base of pectoral- and pelvic-fin spines.

Lips papillose, forming oral disk less than half width of head. Maxillary barbels long (reaching past posterior edge of lower lip when extended posteriorly), thin, and pointed; barbel rugose proximally, almost smooth distally.

Median plates 24; mid-dorsal plates 22–24 (24 in holotype, mode 23); mid-ventral plates 24; rows of plates on caudal peduncle five. Dentary teeth two to three (two in holotype), teeth long and wide; premaxillary teeth four to seven (six in holotype), thinner and shorter than dentary teeth. In new teeth, medial cusps longer and wider than lateral cusps; in worn teeth, medial and lateral cusps of approximately the same length and width. Central buccal papilla absent.

**Color.** Specimens preserved in 70% alcohol with tan base color and small brown spots. Tan base coloration largely uniform, with paler region between orbits, posterior to orbit, and extending posterior to cleithrum. Naked areas white, including ventral surface and areas surrounding origins of pectoral- and pelvic-fin spines. Small brown spots evenly spaced on head. Brown spots on body larger than spots on head, increasing slightly and gradually in size posteriorly. Brown spots on body more closely spaced anteriorly and more distantly spaced posteriorly; on imaginary vertical-oblique lines through the spots, four to five spots occurring per line anteriorly diminishing to two spots per line posteriorly. No spots ventral to an imaginary line between origin of pelvic-fin spine and origin of dorsal caudal-fin spine. Fin rays tan, fin membranes of paired fins and anal fins hyaline. Dorsal-fin rays tan; dorsal-fin membrane tan at base, gradually fading to dark brown band at the distal edge. Caudal-fin membrane on lower lobe transitioning to brown posteriorly. Dark spots present on pectoral-fin rays, pelvic-fin rays, and dorsal-fin rays. Faint white, round spots on dorsal and caudal fins; on dorsal fin, white color more apparent on brown fin membranes than on rays. Odontodes on cheek and fin spines straw-colored with dark brown tips.

**Figure 1.**
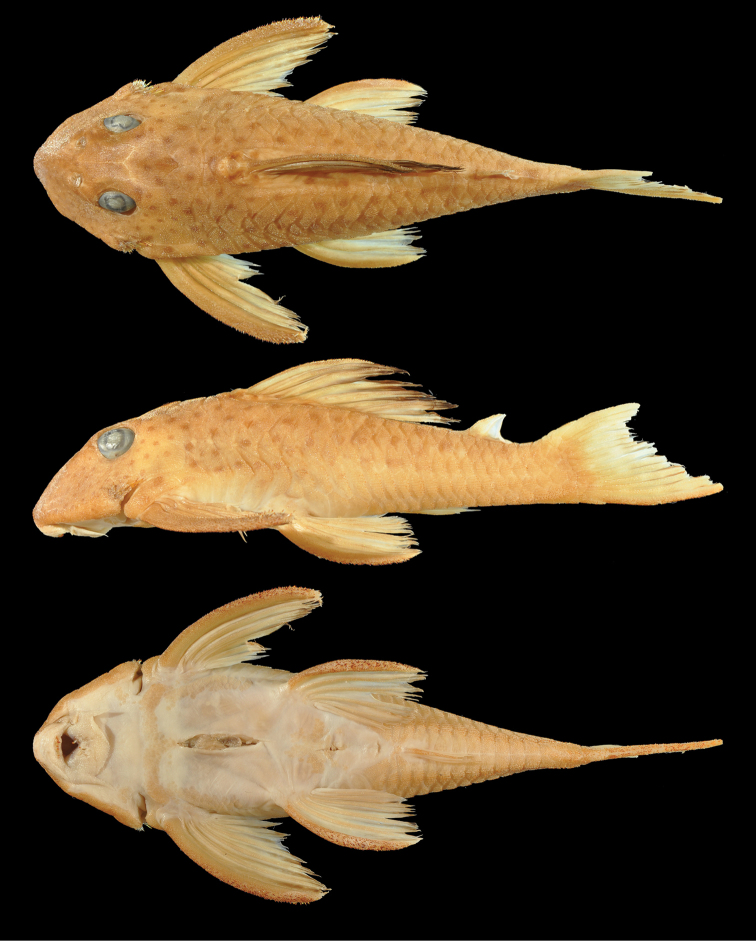
*Hypancistrus phantasma* sp. n., holotype, 123.3 mm SL, dorsal, lateral, and ventral views, MZUSP 116531, Rio Uaupes. Photographs by M Tan.

#### Range.

**Figure 2.**
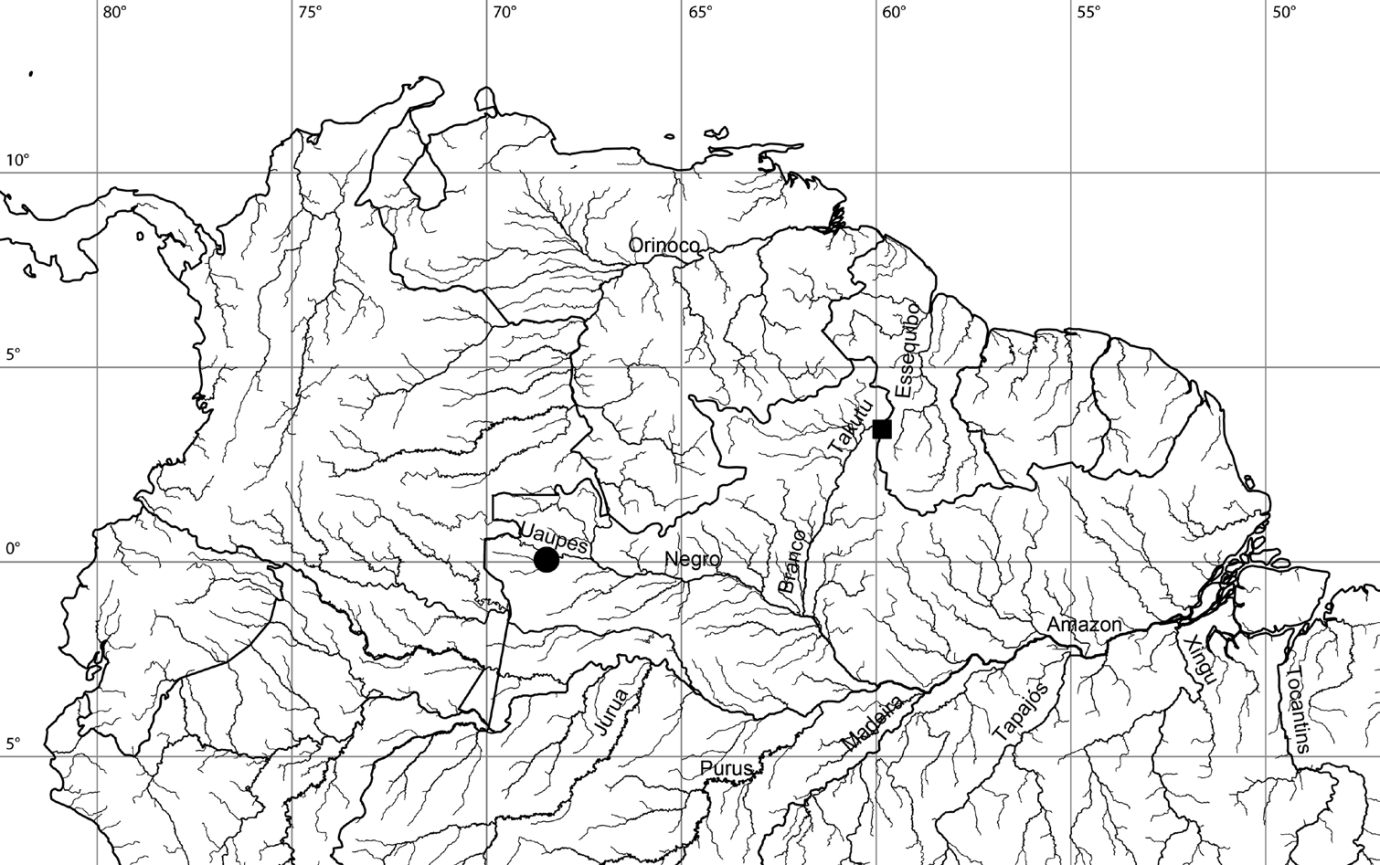
Map of South America, with known localities for *Hypancistrus phantasma* sp. n. (●) and *Hypancistrus margaritatus* sp. n. (■).

Only known from Taracuá of the Rio Uaupes, a tributary of the Rio Negro drainage (Fig. 2).

#### Etymology.

The specific epithet *phantasma* is Latin for “phantom,” and refers to its elusiveness (the described specimens represent the only known specimens, despite nearly a century passing since their collection) and its pale body coloration. It is treated as a noun in apposition.

### Hypancistrus margaritatus sp. n.

Taxon classificationAnimaliaSiluriformesLoricariidae

http://zoobank.org/03E1C489-F788-4E9A-A749-DAFD65D42530

Figures 3 , 4

#### Holotype.

CSBD F1701/AUM 35610 (dual-accessioned), 45.6 mm SL, Amazon Basin, Rio Negro drainage: Rio Branco, Takutu River. Takutu River ca. 2.75 km W Saint Ignatius. Rupununi (Region 9), Guyana, South America. 3.35500°N, 59.83077°W, 5-6 Nov 2002, J.W. Ambruster, M.H. Sabaj, D.C. Werneke, C.L. Allison, M.R. Thomas, C.J. Chin, D. Arjoon, L. Atkinson.

#### Diagnosis.

*Hypancistrus margaritatus* is distinguished from all congeners by its color pattern of dense, small, light-colored spots on a dark base color. Three other described species have a color pattern of light spots on a dark base color: *Hypancistrus inspector*, *Hypancistrus lunaorum*, and *Hypancistrus contradens*. *Hypancistrus margaritatus* has consistently-sized spots on head and trunk about the size of the nasal aperture (vs. smaller spots on head than trunk in *Hypancistrus inspector*, spots usually smaller than half the nasal diameter in *Hypancistrus lunaorum*, and spots ranging in size from equal to or larger in diameter of nasal aperture in *Hypancistrus contradens*). *Hypancistrus margaritatus* also has more spots, 23 in a lateral, horizontal series from snout tip to base of caudal peduncle 23 in holotype (vs. 8–20 spots in *Hypancistrus contradens*; 8–16 in *Hypancistrus lunaorum*). *Hypancistrus margaritatus* has more densely-packed spots relative to *Hypancistrus lunaorum*, with spots on lateral surface of the body having a minimum interspot to spot diameter ratio ranging from 1.0–1.6 (vs. 1.9–4.4 in *Hypancistrus lunaorum*).

#### Description.

Morphometric data given in Table 1. Holotype 45.6 mm SL. Ventral surface from anus to head lacking plates. Lateral plates unkeeled. Cheek plates with 14 hypertrophied odontodes on one side and 18 on other. Frontal, infraorbitals, nasal, preopercle, compound pterotic, and suprapreopercle supporting odontodes. Small ridge of opercle exposed with four odontodes.

Caudal fin emarginate, lower lobe longer than upper. Ventral surface flat. Head tall. Snout short. Distance between verticals through tip of snout and anterior margin of orbit greater than distance between verticals through anterior margin of orbit and posteromedial tip of supraoccipital. Head with steep angle in profile, roughly 45º, from tip of snout to anterior margin of eye. Body depth increases gradually from anterior margin of the eye to dorsal-fin origin, then decreases gradually from dorsal-fin origin to insertion of adipose fin, then caudal peduncle depth increases slightly from insertion of adipose fin to caudal-fin origin.

Supraorbital crest pronounced. Orbital opening oriented at less than 45° from sagittal plane. Interorbital isthmus between supraorbital crests flat. Supraoccipital crest low. Eye large with iris operculum. Gill opening restricted in *L*-conformation, with half of opening vertical and opening laterally and half of opening horizontal and opening ventrally.

Dorsal-fin spine short; depressed dorsal fin reaching slightly beyond pre-adipose plate to origin of adipose fin. Depressed pectoral-fin spine reaching beyond base of pelvic-fin rays; depressed pelvic-fin spine reaching beyond posterior insertion of anal fin. Dorsal fin II,7; caudal fin I,14,I; four dorsal procurrent caudal-fin spines; four ventral procurrent caudal-fin spines; anal fin i4; pectoral fin I,6; pelvic fin i,5. Fin spines and rays supporting odontodes.

Lips papillose, forming oral disk approximately half the width of the head. Maxillary barbels long, not reaching past the posterior edge of the lower lip when extended posteriorly, thin, and pointed; barbel rugose proximally, almost smooth distally.

Median plates 24; mid-dorsal plates 23; mid-ventral plates 24; rows of plates on caudal peduncle five. Dentary teeth five on one side and six on other, long and wide. Premaxillary teeth eight, smaller than dentary teeth. Medial cusps longer and wider than lateral cusps, with cusps separate and angled away from one another (vs. parallel and adjacent). Central buccal papilla absent.

**Figure 3.**
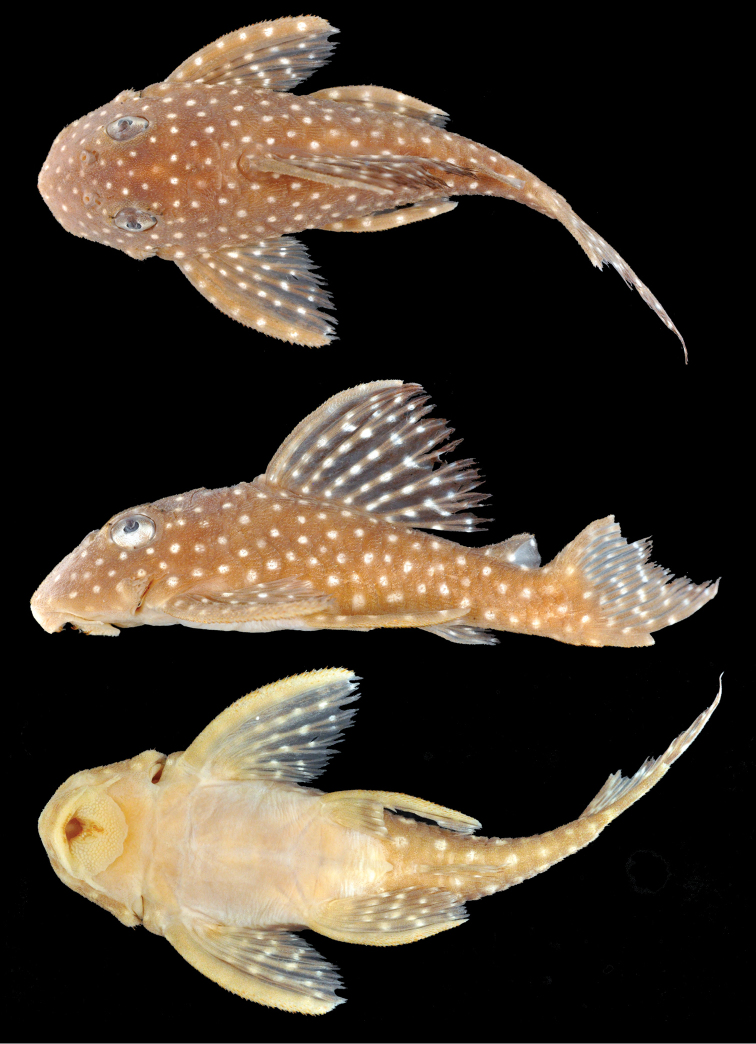
*Hypancistrus margaritatus* sp. n., holotype, 45.6 mm SL, dorsal, lateral, and ventral views, AUM 35610, Takutu River. Photographs by M Tan.

**Color.** Light yellow spots on dark brown base color in life (Figure 3). Brownish-gray base color with small white spots in 70% ethanol. Gray base color mostly uniform, with slightly paler saddle areas at origin of dorsal fin, in middle area of dorsal fin, between dorsal fin and adipose fin, and posterior to adipose fin extending onto dorsal procurrent caudal fin rays. Light spots evenly-sized across body, approximately size of nasal aperture, and smaller in diameter than the length of lateral plates. Light spots relatively evenly spaced and present on all plated regions of body, usually with minimum interspot distance 1.0-1.6 times spot diameter. 21 spots in horizontal series from snout tip to end of caudal peduncle, 23 spots in series along sagittal plane from snout tip to dorsal procurrent caudal fin rays. Dark background color and evenly spaced small white spots also on fin spines and rays; fin-ray membranes hyaline but dusky with sparse melanophores. Six white spots on pectoral-fin spine, decreasing gradually in number to two to three on shortest fin rays. Four to five white spots on pelvic-fin spine, decreasing in number to three spots on shortest fin rays. Spots on dorsal-fin spine and rays in rows roughly parallel to slope of dorsal surface of body between dorsal-fin origin and insertion. Five to six spots on dorsal-fin spine and anterior rays, decreasing in number to three on last dorsal-fin rays. Posterior margin of dorsal fin darker gray than rest of the dorsal fin. Adipose-fin spine with a single spot. Adipose-fin membrane with two small spots. Adipose-fin membrane hyaline, more transparent posteriorly. Ventral naked area posterior to pectoral fin insertion and anterior to anus pale yellow. Mouth and ventral area anterior to pectoral fin insertion yellowish. Eye color dark dorsally with dense melanophores and small white spots, white ventrally with dark shaded spots of sparse melanophores. Odontodes present on fin spines and cheeks straw-colored with dark brown tips.

**Figure 4.**
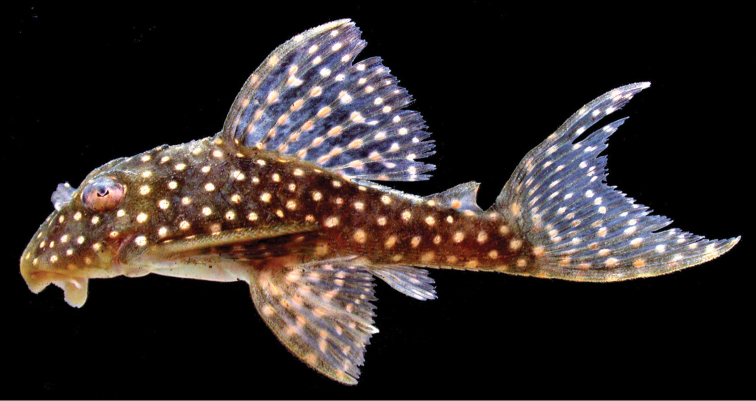
Photograph of live *Hypancistrus margaritatus* holotype, AUM 35610, Takutu River. Image flipped horizontally. Photograph by MH Sabaj Pérez.

#### Range.

*Hypancistrus margaritatus* is described from a single specimen collected in the Takutu River, a tributary of the Rio Branco of the Rio Negro drainage.

#### Etymology.

The specific epithet is Latin for “adorned with pearls,” referring to the dense white spots on the body.

#### Remarks.

The holotype and only known specimen of the species was dual-accessioned at AUM and CSBD because the specimen will be kept at AUM for the near term, but will eventually be repatriated to Guyana.

A *Hypancistrus* from the Takutu River that may represent *Hypancistrus margaritatus* has been designated as L404 in the aquarium pet trade (Stawikowski 2006) and is uncommonly sold (MT *pers. obs.*). L404 appears to be conspecific with *Hypancistrus margaritatus*. In designating the L-number L404, Stawikowski (2006) noted the largest imported specimens were 11 cm in length. The pictured invidual may be larger than the holotype of *Hypancistrus margaritatus*. It also has many more spots (~35 spots from snout tip to caudal peduncle vs. 21 in the holotype of *Hypancistrus margaritatus*). An image of L404 was also provided in Seidel (2008). The image shows approximately 28 spots from snout tip to caudal peduncle, intermediate between that of the holotype of *Hypancistrus margaritatus* and the Stawikowski (2006) image. If L404 is *Hypancistrus margaritatus*, spot number may increase with body size in this species, while the relative distance between spots does not increase.

*Hypancistrus margaritatus* appears to be rare, as only one specimen was collected during four expeditions that yielded 55,156 fish specimens (de Souza et al. 2012). The specimen was collected from within a hole in a lateritic boulder in a run of the mainstem Takutu River. This species is listed as *Hypancistrus* sp. in de Souza et al. (2012).

## Discussion

Of the described species, *Hypancistrus phantasma* appears most similar to *Hypancistrus inspector*, as both species have large maximum sizes relative to other *Hypancistrus* species and share the presence of dark distal edges on the dorsal and caudal fins. *Hypancistrus inspector* is found in the upper Rio Negro, and *Hypancistrus phantasma* is found in the Rio Uaupes, a tributary to the Rio Negro. Because of their morphological similarity and close geographical proximity, *Hypancistrus inspector* and *Hypancistrus phantasma* may be closely related. They can be separated by the light base color and presence of dark spots in *Hypancistrus phantasma* vs. dark base color with light spots in *Hypancistrus inspector*. Although *Hypancistrus inspector* is distinguished from *Hypancistrus contradens* in part by having a dorsal fin that does not reach the adipose fin when depressed (Armbruster et al. 2007), the dorsal fin of *Hypancistrus phantasma* does usually reach the preadipose plate. Also, *Hypancistrus phantasma* has significantly fewer dentary teeth, two to three vs. five to 10 in *Hypancistrus inspector*; however, gaps between the dentary teeth in *Hypancistrus phantasma* specimens examined suggest that they may have lost teeth. A color pattern of a pale base color with black spots is found in an undescribed species of *Hypancistrus* from the Rio Xingu referred to as L174 (Seidel 2008). Based on photographs and reported information, this species differs from *Hypancistrus phantasma* in its smaller maximum body size, absence of a black edge on the dorsal and caudal fins, and black spots being large and sometimes near enough to coalesce, particularly behind the head where they may form a collar.

The specimens of *Hypancistrus phantasma* were collected in 1924, and it is likely their coloration has been affected by age since preservation. Nevertheless, the distal edge of the dorsal fin is relatively dark. Faded specimens of *Hypancistrus inspector* do not have dark spots like those in *Hypancistrus phantasma*, and merely show a reduction in intensity of coloration. Thus, even if the preserved color pattern in *Hypancistrus phantasma* may not be representative of the live coloration, the presence of small dark spots on the body is diagnostic.

*Hypancistrus margaritatus* is most similar to the other small-bodied, spotted *Hypancistrus*: *Hypancistrus contradens* and *Hypancistrus lunaorum*. It differs from these species in having more body spots. In *Hypancistrus contradens* and *Hypancistrus lunaorum*, the spots increase in number allometrically (Armbruster et al. 2007), but the spots never reach the number present in *Hypancistrus margaritatus*. It appears there is an allometric relationship with spot number and body size in *Hypancistrus margaritatus* as well, based on images of specimens identified as L404 in aquarium literature presumed to be conspecific. Specimens of *Hypancistrus contradens* and *Hypancistrus lunaorum* at equivalent body sizes to the *Hypancistrus margaritatus* holotype have far fewer spots, so a larger number of spots in *Hypancistrus margaritatus* likely distinguishes this species from *Hypancistrus contradens* and *Hypancistrus lunaorum* across their size ranges. In addition, the distance between spots in *Hypancistrus lunaorum* relative to spot size is much greater than in *Hypancistrus margaritatus*, *Hypancistrus contradens*, and *Hypancistrus inspector*.

*Hypancistrus margaritatus* is also similar to some undescribed forms of *Hypancistrus*. A specimen of *Hypancistrus* with small spots from the Rio Madeira was identified as Hypancistrus cf. inspector (de Queiroz et al. 2013). The photograph shows a specimen that is heavily damaged, but has many small spots like *Hypancistrus margaritatus*. The spots are more densely packed (minimum interspot distance < spot diameter) than in *Hypancistrus margaritatus*. There do not appear to be spots on the dorsal and caudal fins, which differs from the presence of spots on these fins in *Hypancistrus margaritatus*, *Hypancistrus contradens*, *Hypancistrus inspector*, and *Hypancistrus lunaorum*. The specimen likely represents another undescribed species. In the aquarium literature, *Hypancistrus margaritatus* (as L404) has been described as similar to another *Hypancistrus* form called L136, which also has a color pattern of dense, light-colored spots on a dark base color (Stawikowski 2006). The identity of these forms and their possible conspecificity with *Hypancistrus margaritatus* should be assessed. Another spotted L-number, L004 (and putative conspecific L-numbers L005, L028, L073), appears similar to *Hypancistrus lunaorum*, possessing small spots (smaller in diameter than nasal aperture) with a larger interspot distance-spot diameter ratio (Seidel 2008). The spotted L262 appears to have smaller spots and in higher numbers than *Hypancistrus margaritatus* (Seidel 2008).

Both new species of *Hypancistrus* may be extremely rare. *Hypancistrus margaritatus* was only collected once, as a single specimen, in four collecting trips to the Takutu River region (de Souza et al. 2012). However, this species has apparently been collected occasionally by aquarium hobbyists and designated as L404 (Stawikowski 2006). To the knowledge of the authors, *Hypancistrus phantasma* has not been collected since 1924. There are insufficient data to assess the conservation statuses of these species.

### Other materials examined

Locality data listed in Armbruster (2002), Armbruster et al. (2007).

*Hypancistrus contradens*: AUM 37978, AUM 42097, AUM 42170, AUM 42190, AUM 54471

*Hypancistrus inspector*: AUM 31019, AUM 39234, AUM 42198, MCNG 12133, MCNG 37040

*Hypancistrus lunaorum*: AUM 39247, AUM 39277, AUM 39590, AUM 39837, AUM 42120, AUM 42142, AUM 44315

## Supplementary Material

XML Treatment for Hypancistrus phantasma

XML Treatment for Hypancistrus margaritatus
